# Clinical, environmental, and behavioral characteristics associated with *Cryptosporidium* infection among children with moderate-to-severe diarrhea in rural western Kenya, 2008–2012: The Global Enteric Multicenter Study (GEMS)

**DOI:** 10.1371/journal.pntd.0006640

**Published:** 2018-07-12

**Authors:** Miranda J. Delahoy, Richard Omore, Tracy L. Ayers, Katharine A. Schilling, Anna J. Blackstock, J. Benjamin Ochieng, Feny Moke, Peter Jaron, Alex Awuor, Caleb Okonji, Jane Juma, Tamer H. Farag, Dilruba Nasrin, Sandra Panchalingam, James P. Nataro, Karen L. Kotloff, Myron M. Levine, Joseph Oundo, Dawn M. Roellig, Lihua Xiao, Michele B. Parsons, Kayla Laserson, Eric D. Mintz, Robert F. Breiman, Ciara E. O'Reilly

**Affiliations:** 1 National Center for Emerging and Zoonotic Infectious Diseases, Centers for Disease Control and Prevention (CDC), Atlanta, Georgia, United States of America; 2 Department of Environmental Health, Rollins School of Public Health, Emory University, Atlanta, Georgia, United States of America; 3 Kenya Medical Research Institute/CDC, Kisumu, Kenya; 4 Centre for Global Health Research, Kenya Medical Research Institute, Kisumu, Kenya; 5 Center for Vaccine Development, University of Maryland School of Medicine, Baltimore, Maryland, United States of America; 6 Institute for Health Metrics and Evaluation, University of Washington, Seattle, Washington, United States of America; 7 Department of Pediatrics, University of Virginia School of Medicine, Charlottesville, Virginia, United States of America; 8 CDC-Kenya, Nairobi, Kenya; 9 Center for Global Health, Centers for Disease Control and Prevention, Atlanta, Georgia, United States of America; 10 CDC-India, Delhi, India; 11 Emory Global Health Institute, Emory University, Atlanta, Georgia, United States of America; Christian Medical College, INDIA

## Abstract

**Background:**

*Cryptosporidium* is a leading cause of moderate-to-severe diarrhea (MSD) in young children in Africa. We examined factors associated with *Cryptosporidium* infection in MSD cases enrolled at the rural western Kenya Global Enteric Multicenter Study (GEMS) site from 2008-2012.

**Methodology/Principal findings:**

At health facility enrollment, stool samples were tested for enteric pathogens and data on clinical, environmental, and behavioral characteristics collected. Each child’s health status was recorded at 60-day follow-up. Data were analyzed using logistic regression. Of the 1,778 children with MSD enrolled as cases in the GEMS-Kenya case-control study, 11% had *Cryptosporidium* detected in stool by enzyme immunoassay; in a genotyped subset, 81% were *C*. *hominis*. Among MSD cases, being an infant, having mucus in stool, and having prolonged/persistent duration diarrhea were associated with being *Cryptosporidium-*positive. Both boiling drinking water and using rainwater as the main drinking water source were protective factors for being *Cryptosporidium-*positive. At follow-up, *Cryptosporidium*-positive cases had increased odds of being stunted (adjusted odds ratio [aOR] = 1.65, 95% CI: 1.06–2.57), underweight (aOR = 2.08, 95% CI: 1.34–3.22), or wasted (aOR = 2.04, 95% CI: 1.21–3.43), and had significantly larger negative changes in height- and weight-for-age z-scores from enrollment.

**Conclusions/Significance:**

*Cryptosporidium* contributes significantly to diarrheal illness in young children in western Kenya. Advances in point of care detection, prevention/control approaches, effective water treatment technologies, and clinical management options for children with cryptosporidiosis are needed.

## Author summary

*Cryptosporidium* is an important cause of childhood diarrhea. Research on cryptosporidiosis in countries where it is endemic remains limited; few studies have comprehensively examined risk factors for children in Kenya and similar settings. We examined characteristics associated with *Cryptosporidium* in children with moderate-to-severe diarrhea in rural western Kenya. We found there is little to clinically distinguish cryptosporidiosis from other childhood diarrhea in the absence of point of care diagnostics. Infants had the highest odds of *Cryptosporidium* infection; it has been previously established that *Cryptosporidium* infections in infancy can have severe consequences. Prolonged/persistent duration diarrhea and growth shortfalls were significantly more pronounced among cases with *Cryptosporidium*. Undernutrition and stunting in children in low- and middle-income countries have predicted decreased cognitive and school performance, thus long-term consequences could be appreciable. Using rainwater as the primary drinking water source and boiling drinking water were protective against *Cryptosporidium* infection, thus certain water sources may contribute to transmission. Like other studies in Kenya, we predominantly identified *Cryptosporidium hominis*, an anthropogenic species. Advances in point of care detection, prevention and control approaches, effective water treatment technologies, and clinical management options are needed to mitigate the potentially severe and long-term consequences of *Cryptosporidium* infection in children.

## Introduction

The Global Enteric Multicenter Study (GEMS) was undertaken to assess the burden and etiology of moderate-to-severe diarrhea (MSD) in seven countries, three in South Asia and four in sub-Saharan Africa. In all African sites, *Cryptosporidium* was the second-highest enteric pathogen attributable to infant MSD; in GEMS Kenya, *Cryptosporidium* was a major pathogen across all age groups (0–11, 12–23, and 24–59 months) [1]. *Cryptosporidium* was also identified as one of five pathogens with the highest attributable burden of infant diarrhea in a study of malnutrition and enteric disease (MAL-ED), a cohort study that compared diarrheal and non-diarrheal stools in children under two years old collected at community surveillance visits at 8 sites in South America, Africa, and Asia [2]. Based on GEMS data, it has been estimated that there are nearly three million annual diarrhea episodes attributable to *Cryptosporidium* in young children in sub-Saharan Africa [3]. Globally, acute *Cryptosporidium* infections are estimated to cause 48,000 annual deaths in children under five years old [4].

*Cryptosporidium* infections in young children in low- and middle-income countries have been associated with excess mortality [5], an excess burden of diarrhea later in life [6], and growth faltering, the deficits of which may not be recovered for those children infected during infancy [7]. *Cryptosporidium* has been associated with decreases in height-for-age z-scores in children, even in the absence of diarrhea symptoms [4]. *Cryptosporidium* infections have been associated with persistent diarrhea in Kenya [8,9]. *Cryptosporidium* is highly tolerant to disinfection with chlorine [10].

Nitazoxanide can treat cryptosporidiosis in immunocompetent children 1–11 years old [11]; however, it is not often available in developing countries [12] and is presently not approved for infants [11]. There is currently no vaccine available for *Cryptosporidium*; however, the evidence of acquired immunity suggests that one could be effective [12].

Although outbreaks of *Cryptosporidium* in developed countries have been studied in detail, less is known about risk factors for cryptosporidiosis in countries where it is endemic [10]. Reviews of risk factors for *Cryptosporidium* infection identified malnutrition, contact with domestic animals, non-exclusive breastfeeding in infants, lack of sanitation facilities, and crowded living conditions as possible risk factors for infection in low- and middle-income countries [13,14]. Few studies have examined risk factors for *Cryptosporidium* infection in Kenyan children [15–17]. In Kenya, risk factors for *Cryptosporidium* in children include being HIV-positive [17], or having an HIV-positive mother [15].

We describe the prevalence of *Cryptosporidium* infections in Kenyan children under five years old with MSD, assess clinical, environmental, and behavioral characteristics associated with *Cryptosporidium* infection, and describe the outcomes and consequences of cryptosporidiosis.

## Methods

### Global Enteric Multicenter Study (GEMS)

We evaluated data collected in Kenya from cases enrolled in GEMS, a four-year, prospective, age-stratified, health facility-based matched case-control study of MSD among children aged 0–59 months residing within a defined and enumerated population. The rationale, study design, clinical and microbiologic methods, and assumptions of GEMS have been described elsewhere [18,19]. Briefly, GEMS enrolled MSD cases from selected sentinel health facilities in each of three age strata (0-11, 12-23, and 24-59 months old), along with 1–3 matched community controls who had not had diarrhea in the week before enrollment. MSD was defined as having three or more loose stools in the previous 24 hours, with onset within the 7 days prior to enrollment, and having one or more of the following illness severity characteristics: loss of skin turgor, sunken eyes, required intravenous fluid rehydration, dysentery (blood in stool), or required hospitalization [18].

At enrollment, demographic, clinical, epidemiological information, and stool samples were collected. *Cryptosporidium* oocyst antigens were detected in whole stool specimens by enzyme immunoassay (EIA; TechLab, Inc, VA, USA). Detailed laboratory methods are described elsewhere [19]. DNA was extracted from a subset of stools that were EIA-positive for *Cryptosporidium*. Restriction fragment length polymorphism analyses and DNA sequencing of polymerase chain reaction (PCR) products were used to identify *Cryptosporidium* genotypes for these specimens at the U.S. Centers for Disease Control and Prevention (CDC) [20].

To assess each child’s health status, a home visit including focused physical exams and anthropometric measurements was conducted ~60 days (acceptable range 50–90 days) following enrollment. Mortality that occurred at any time between enrollment and this follow-up was recorded.

### Study site

In Kenya, children were enrolled between January 31, 2008 and January 29, 2011, and again from October 31, 2011 to September 30, 2012. The study was conducted in Siaya County, in the areas of Gem and Asembo, and during the second enrollment period, in the areas of Asembo and Karemo due to the Kenya Medical Research Institute (KEMRI)/CDC health and demographic surveillance system moving activities. This health and demographic surveillance system has been operating in these communities since 2001. The study setting has high rates of child mortality, malaria, HIV, and tuberculosis, and has been described elsewhere [21,22].

### Study definitions

#### Days of diarrhea

At enrollment caretakers were (1) asked how many days the case child had been experiencing diarrhea in the week before presenting at the health facility and (2) instructed on how to record the presence/absence of diarrhea for 14 days following enrollment on a Memory Aid form [18]. The total number of days of diarrhea was calculated as the sum of these durations, with a maximum of 21 possible diarrhea days, which may include multiple episodes [8]. *Acute diarrhea* was defined as having 1–6 days of diarrhea in the 21-day period; having 7–13 and ≥14 days refer to *prolonged* and *persistent diarrhea*, respectively.

#### Anthropometry measurements

Height and weight for each child were measured at enrollment and at 60-day follow-up; details on measurement methods are described elsewhere [18]. Height/length-for-age, weight-for-age, and weight-for-height/length z-scores (HAZ, WAZ, and WHZ) were calculated using a WHO SAS macro with the WHO Child Growth Standards as the reference population [23,24]. For case children requiring rehydration, weight measurements after rehydration were used. If the child was under observation for more than four hours, weight measurements were repeated upon discharge from the health facility. To account for rehydration, the last recorded weight measurement was used as the baseline value. Stunted, underweight, and wasted were defined as having HAZ<-2, WAZ<-2, and WHZ<-2 respectively. Children with HAZ<-3, WAZ<-3, or WHZ<-3 were considered severely malnourished. All anthropometric measures identified as extreme measures using the WHO definition (|HAZ|>6, WAZ<-6, WAZ>5, or |WHZ|>5) were excluded [23], as were outliers identified using median absolute deviation methods [25].

#### HIV testing

A subset of cases and their biological mothers had HIV status captured. Children ≥18 months old had a rapid HIV antibody test and children <18 months old had a confirmatory PCR test. Persons identified as HIV positive were referred to care and treatment.

#### Water definitions

Surface water was defined as drinking water coming from a pond, lake, river, stream, or dam, as is standard practice [26], or water coming from an earth pan/water pan, which is western Kenya specific. Standard definitions were used to define improved and unimproved water sources [26].

#### Breastfeeding definitions

*Partial breastfeeding* refers to providing children with breast milk and supplementing with other food or liquid, whereas *exclusive breastfeeding* refers to providing children only breast milk without supplementation.

### Statistical analysis

Analyses were performed in SAS 9.4 (SAS Institute, Inc., Cary, NC) and R 3.4.0 (R Foundation for Statistical Computing, Vienna, Austria). To assess variables associated with *Cryptosporidium* positivity, univariable logistic regression models were used to compute odds ratios (ORs) and 95% confidence intervals (CIs). Since *Cryptosporidium* risk factors may be modified by age and the sample size might limit detection of interactions, we assessed for effect modification by age category for all variables in separate models with a *p*<0.05 cutoff for significance. To assess whether each risk factor was confounded by socioeconomic status (SES) we ran models with and without SES and considered confounding if effect sizes changed >10%.

Two multivariable models were generated to identify clinical, demographic, environmental, and behavioral characteristics associated with being *Cryptosporidium*-positive. The first model examined the clinical presentation at enrollment of case children, including age strata and sex (see Table 1) and all variables in Table 2 (except duration of diarrhea, which includes information collected post-enrollment). The second model sought to identify demographic, environmental, and behavioral characteristics that may be risk factors for *Cryptosporidium* infection (all variables in Table 1, and the following caretaker-reported water, sanitation, and hygiene characteristics collected at enrollment: primary source of drinking water, whether water was always available from the main drinking water source, whether the child was given stored water in the two weeks prior to enrollment, whether the caretaker boiled or filtered drinking water, whether there was a facility for feces disposal, whether the caretaker uses soap when washing hands, and whether the caretaker washes their hands at the following times: before eating, after defecating, before nursing, before cooking, after cleaning a child, and after touching an animal). Breastfeeding was not considered in either model as collinearity with age was identified, and information on breastfeeding is only available for children under two years old in the first three years of GEMS (n = 1,083); questions on breastfeeding changed during the fourth study year. Model selection was performed using the Least Absolute Shrinkage and Selection Operator (LASSO) method using the minimum error lambda [27,28]. While LASSO methods were used to identify variables for inclusion, parameter estimates and CIs are derived by standard logistic regression maximum likelihood methods.

**Table 1:** Demographic and household characteristics of GEMS-Kenya cases (N = 1,778) by *Cryptosporidium* status, western Kenya, 2008–2012.

	*Cryptosporidium-* positive cases (N = 195)	*Cryptosporidium-* negative cases (N = 1,583)	OR (95% CI)
Age category			
0–11 months	119 (61.0%)	710 (44.9%)	**3.32 (2.08, 5.31)**
12–23 months	54 (27.7%)	437 (27.6%)	1.36 (0.96, 1.91)
24–59 months	22 (11.3%)	436 (27.5%)	ref.
Male sex	120 (61.5%)	890 (56.2%)	1.25 (0.92, 1.69)
Caretaker completed primary school	96 (49.2%)	700 (44.2%)	1.22 (0.91, 1.65)
People sleeping in house^a^ (median)	4 (IQR: 4–5)	4 (IQR: 4–5)	
Above median^b^	82 (42.1%)	737 (46.6%)	0.83 (0.62, 1.12)
Young children living in house^c^ (median)	2 (IQR: 1–2)	2 (IQR: 1–2)	
Above median^b^	30 (15.4%)	173 (10.9%)	1.48 (0.96, 2.23)
Household has agricultural land	173 (88.7%)	1,438 (90.8%)	0.79 (0.50, 1.31)

OR = odds ratio (logistic regression); CI = confidence interval; ref. = referent group; IQR = interquartile range; **bolding** indicates statistically significant at *p*<0.05

a. Caretaker response when asked “how many people have been sleeping regularly in your household for the past 6 months?”

b. Greater than the median value for all GEMS-Kenya cases combined

c. Young children are defined as children under five years old

**Table 2:** Clinical presentation of GEMS-Kenya cases (N = 1,778) by *Cryptosporidium* status, western Kenya, 2008–2012.

	*Cryptosporidium-* positive cases (N = 195)	*Cryptosporidium-* negative cases (N = 1,583)	OR (95% CI)
**Measured or observed by clinician at enrollment**			
Had fever^a^ at enrollment	50 (25.6%)	495 (31.3%)	0.76 (0.54, 1.06)
Required intravenous rehydration	33 (16.9%)	203 (12.8%)	1.38 (0.91, 2.05)
Child’s mouth:			
Normal	6 (3.1%)	63 (4.0%)	ref.
Somewhat dry	148 (75.9%)	1,226 (77.4%)	1.27 (0.58, 3.32)
Very dry	41 (21.0%)	293 (18.5%)	1.47 (0.64, 3.99)
Child’s mental state:			
Normal	64 (32.8%)	565 (35.7%)	ref.
Irritable or restless	125 (64.1%)	975 (61.6%)	1.13 (0.83, 1.56)
Lethargic or unconscious	6 (3.1%)	43 (2.7%)	1.23 (0.46, 2.80)
**Reported by caretaker at enrollment**			
Child is very thirsty	153 (78.5%)	1,218 (76.9%)	1.04 (0.73, 1.51)
Child drinks poorly or is unable to drink	32 (16.4%)	256 (16.2%)	1.02 (0.67, 1.50)
Child has wrinkled skin	39 (20.0%)	256 (16.2%)	1.29 (0.87, 1.85)
Child has fast breathing	27 (13.8%)	260 (16.4%)	0.82 (0.52, 1.23)
Maximum # of stools child passed in a 24-hour period during illness up to enrollment			
≤6	145 (74.4%)	1,174 (74.2%)	ref.
7–10	42 (21.5%)	356 (22.5%)	0.96 (0.66, 1.36)
>10	8 (4.1%)	53 (3.3%)	1.22 (0.53, 2.48)
**Characteristics of stool sample provided at enrollment**			
Stool sample watery^b^	114 (58.5%)	817 (51.6%)	1.32 (0.98, 1.79)
Blood in stool sample	6 (3.1%)	60 (3.8%)	0.81 (0.31, 1.75)
Pus in stool sample	7 (3.6%)	48 (3.0%)	1.19 (0.49, 2.50)
Mucus in stool sample	155 (79.5%)	1,096 (69.2%)	**1.72 (1.21, 2.51)**
**Diarrhea duration**^**c**^	(N = 169)^d^	(N = 1,401)^d^	
Acute diarrhea (1–6 days)	57 (33.7%)	680 (48.5%)	ref.
Prolonged diarrhea (7–13 days)	91 (53.8%)	648 (46.3%)	**1.68 (1.18, 2.37)**
Persistent diarrhea (≥14 days)	21 (12.4%)	73 (5.2%)	**3.43 (1.97, 5.98)**

OR = odds ratio (logistic regression); CI = confidence interval; ref. = referent group; **bolding** indicates statistically significant at *p*<0.05

a. Temperature >38°C measured in health facility

b. Compared to stool that was formed, soft, or thick liquid

c. At enrollment caretakers were (1) asked how many days the case child had been experiencing diarrhea in the week before presenting at the health facility and (2) instructed on how to record the presence/absence of diarrhea for 14 days following enrollment on a pre-piloted Memory Aid form. The total number of days of diarrhea was calculated as the sum of these durations, with a maximum of 21 possible diarrhea days, which may include multiple episodes.

d. Diarrhea duration is only reported for case children with complete Memory Aid forms

#### Anthropometry analyses

The proportions of children who were stunted, underweight, and wasted at enrollment and at follow-up were compared for *Cryptosporidium*-positive and *Cryptosporidium*-negative cases using ORs and 95% CIs. Models with follow-up anthropometry measures as outcomes controlled for baseline measures. Changes in HAZ, WAZ, and WHZ were calculated by subtracting the baseline measure from the follow-up measure. A Wilcoxon rank sum test was used to evaluate differences in distributions of z-scores for these changes for *Cryptosporidium-*positive and *Cryptosporidium-*negative cases. Only those with follow-up measurements within an acceptable range of 50–90 days were included.

For anthropometry analyses, effect modification by age and sex was assessed by including interaction terms one-by-one into the models. If interaction could be assessed (*i*.*e*., the model converged) and the interaction was significant at alpha<0.05, results were stratified accordingly.

#### Sensitivity analyses

Children could be re-enrolled as a case 90 days post-enrollment. Children who were enrolled more than once as a case into GEMS Kenya were included in all analyses, as were children with multiple enteric pathogens detected in stool. To assess bias in this approach, two sensitivity analyses were performed. Models were re-run on the subset of case children (n = 1,483) who were only enrolled as a case once. We also compared *Cryptosporidium*-positive cases with a single pathogen to *Cryptosporidium*-positive cases with multiple enteric pathogens (S1 and S2 Tables).

### Ethical review

Written informed consent was collected from all parents of children who participated in GEMS. The GEMS protocol was approved by the Scientific and Ethical Review Committees of KEMRI (Protocol #1155) and the Institutional Review Board (IRB) of the University of Maryland School of Medicine, Baltimore, MD, USA (UMD Protocol #H-28327). CDC (Atlanta, GA, USA) formally deferred its review to the UMD IRB (CDC Protocol #5038).

## Results

### Demographic and household characteristics

**Fig 1:**
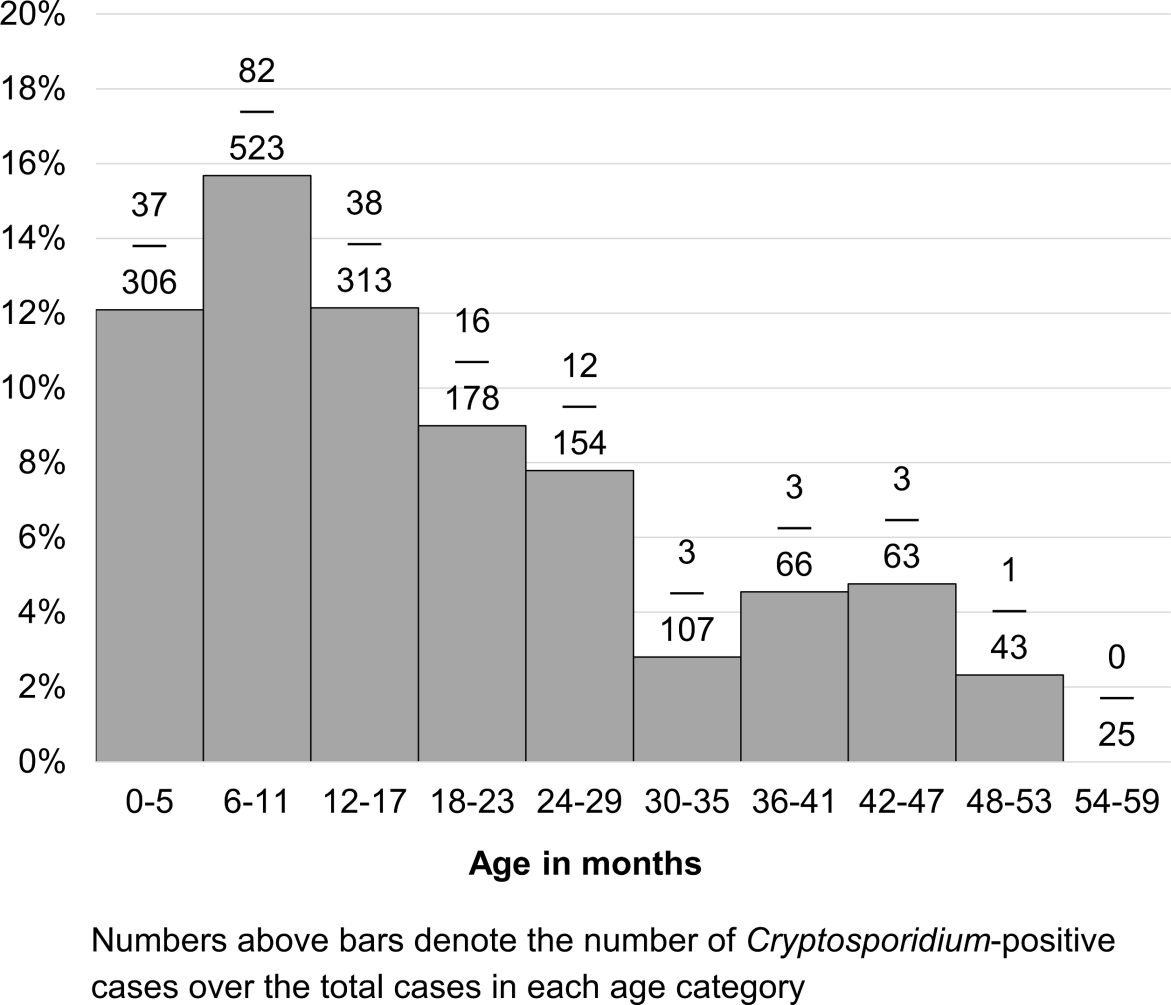
Percent of GEMS-Kenya cases (N = 1,778) with *Cryptosporidium* by age, western Kenya, 2008–2012.

Among the 1,778 MSD case children enrolled, *Cryptosporidium* was identified in 195 cases (11.0%). *Cryptosporidium* infections were more frequently identified in infants (<12 months old), with a peak in *Cryptosporidium* infection at 6–11 months old (Table 1 and Fig 1). Compared to case children aged 24–59 months, infants had over triple the odds of being *Cryptosporidium-*positive (OR = 3.32; 95% CI: 2.08–5.31). Other demographic and household characteristics were similar between *Cryptosporidium*-positive and *Cryptosporidium*-negative cases (Table 1). A non-statistically significant relationship between *Cryptosporidium* status and having agricultural land was confounded by SES. As no other variable was confounded by SES, only unadjusted effect measures are shown in Table 1.

### Clinical presentation and outcomes

The clinical presentation of *Cryptosporidium-*positive and *Cryptosporidium-*negative cases was similar (Table 2). Mucus in the stool was significantly associated with being *Cryptosporidium-*positive (OR = 1.72, 95% CI: 1.21–2.51). Only age category and mucus in stool remained in the final multivariable clinical model (not presented). The findings were the same when children who were enrolled multiple times as a case were excluded. Having mucus in the stool remained significantly associated with *Cryptosporidium* infection controlling for age (aOR = 1.50; 95% CI: 1.05–2.20).

Approximately two-thirds (66%) of *Cryptosporidium-*positive cases with daily information on diarrhea experienced prolonged or persistent diarrhea, compared to approximately half (51%) of *Cryptosporidium-*negative cases (Table 2). Compared to cases experiencing acute diarrhea, cases experiencing prolonged diarrhea were significantly more likely to be *Cryptosporidium*-positive (OR = 1.68; 95% CI: 1.18–2.37); cases experiencing persistent diarrhea were also significantly more likely to be *Cryptosporidium*-positive compared to cases experiencing acute diarrhea (OR = 3.43; 95% CI: 1.97–5.98).

### Indicators of malnutrition

At enrollment, sex was a significant effect modifier of the relationship between *Cryptosporidium* and stunting/severe stunting. Among girls, *Cryptosporidium*-positive cases had significantly greater odds of being stunted at baseline than *Cryptosporidium*-negative cases (OR = 1.82, 95% CI: 1.10–3.01). There were no other statistically significant differences in malnutrition indicators between *Cryptosporidium*-positive and *Cryptosporidium*-negative cases at enrollment (Table 3).

**Table 3:** Anthropometric indicators of malnutrition for GEMS-Kenya cases (N = 1,778), by *Cryptosporidium* status, western Kenya, 2008–2012.

	*Cryptosporidium-* positive cases (N = 195)^a^	*Cryptosporidium-* negative cases (N = 1,583)^a^	aOR^b^ (95% CI)
**HAZ (height-for-age z-scores)**^c^			
Stunted (HAZ<-2) at enrollment			
Boys	29/120 (24.2%)	275/884 (31.1%)	0.71 (0.45, 1.10)
Girls	27/75 (36.0%)	163/691 (23.6%)	**1.82 (1.10, 3.01)**
Stunted (HAZ<-2) at follow-up	70/178 (39.3%)	496/1,468 (33.8%)	**1.65 (1.06, 2.57)**
Boys	38/111 (34.2%)	311/830 (37.5%)	1.26 (0.71, 2.23)
Girls	32/67 (47.8%)	185/638 (29.0%)	**2.49 (1.22, 5.06)**
Severely stunted (HAZ<-3) at enrollment			
Boys	7/120 (5.8%)	96/884 (10.9%)	0.51 (0.23, 1.12)
Girls	11/75 (14.7%)	58/691 (8.4%)	1.88 (0.94, 3.75)
Severely stunted (HAZ<-3) at follow-up	21/178 (11.8%)	184/1,468 (12.5%)	0.85 (0.43, 1.65)
Boys	11/111 (9.9%)	118/830 (14.2%)	0.87 (0.39, 1.94)
Girls	10/67 (14.9%)	66/638 (10.3%)	0.62 (0.17, 2.24)
	Median (IQR)	Median (IQR)	*p*-value^d^
HAZ change (follow-up–baseline)	-0.40 (-0.73, -0.11)	-0.23 (-0.54, 0.00)	**<0.0001**
Boys	-0.33 (-0.73, -0.10)	-0.27 (-0.58, -0.02)	0.0779
Girls	-0.49 (-0.73, -0.17)	-0.19 (-0.48, 0.02)	**0.0001**
**WAZ (weight-for-age z-scores)**			
Underweight (WAZ<-2) at enrollment	43/195 (22.1%)	285/1,575 (18.1%)	1.28 (0.89, 1.84)
Underweight (WAZ<-2) at follow-up	50/177 (28.2%)	263/1,461 (18.0%)	**2.08 (1.34, 3.22)**
Severely underweight (WAZ<-3) at enrollment	17/195 (8.7%)	87/1,575 (5.5%)	1.63 (0.95, 2.81)
Severely underweight (WAZ<-3) at follow-up	17/177 (9.6%)	92/1,461 (6.3%)	1.34 (0.70, 2.55)
	Median (IQR)	Median (IQR)	*p*-value^d^
WAZ change (follow-up–baseline)	-0.25 (-0.62, 0.25)	-0.05 (-0.45, 0.29)	**0.0122**
**WHZ (weight-for-height z-scores)**			
Wasted (WHZ<-2) at enrollment	18/195 (9.2%)	122/1,567 (7.8%)	1.20 (0.72, 2.02)
Wasted (WHZ<-2) at follow-up	22/176 (12.5%)	102/1,457 (7.0%)	**2.04 (1.21, 3.43)**
Severely wasted (WHZ<-3) at enrollment	6/195 (3.1%)	27/1,567 (1.7%)	1.81 (0.74, 4.44)
Severely wasted (WHZ<-3) at follow-up	5/176 (2.8%)	28/1,457 (1.9%)	1.16 (0.41, 3.27)
	Median (IQR)	Median (IQR)	*p*-value^d^
WHZ change (follow-up–baseline)	-0.01 (-0.68, 0.52)	0.08 (-0.48, 0.58)	0.1359

aOR = adjusted odds ratio (logistic regression); CI = confidence interval; IQR = interquartile range; **bolding** = statistically significant at *p*<0.05

Severely stunted, severely underweight, and severely wasted are subsets of stunted, underweight, and wasted, respectively.

a. Actual denominators exclude outliers (+/- 3.5 median absolute deviation or, based on WHO definitions, |HAZ|>6, WAZ<-6, WAZ>5, |WHZ|>5); for follow-up analyses, only those with a follow-up within 50–90 days of enrollment are included.

b. Referent: *Cryptosporidium-*negative GEMS Kenya cases. Adjusted ORs control for baseline status (follow-up analyses of (severe) stunting, (severe) underweight, and (severe) wasting control for being (severely) stunted, (severely) underweight, and (severely) wasted at baseline, respectively).

c. Child’s sex significantly modified the relationship between *Cryptosporidium* and stunting/severe stunting at baseline, but not at follow-up. For consistency, follow-up stunting/severe stunting are also stratified by sex in addition to the aggregate OR. Also for consistency, HAZ change from baseline to follow-up is presented in aggregate and stratified by sex.

d. Difference between *Cryptosporidium-*positive cases and *Cryptosporidium-*negative cases: Wilcoxon rank sum test.

At the 60-day follow-up, *Cryptosporidium-*positive cases had significantly greater odds of being stunted (aOR = 1.65, 95% CI: 1.06–2.57), underweight (aOR = 2.08, 95% CI: 1.34–3.22), or wasted (aOR = 2.04, 95% CI: 1.21–3.43) compared to *Cryptosporidium*-negative cases, controlling for baseline status for each measure (Table 3).

*Cryptosporidium*-positive cases had significantly larger negative changes in HAZ and WAZ measures from baseline to follow-up. When considering HAZ by sex, female *Cryptosporidium*-positive cases had significantly larger negative changes in HAZ compared to female *Cryptosporidium*-negative cases (Table 3).

### HIV status

HIV status was available for 58.8% of GEMS-Kenya cases. Of the 114 *Cryptosporidium-*positive cases with available HIV test results, 5 (4.4%) were HIV-positive, compared to 3.0% (28/932) of *Cryptosporidium-*negative cases (*p* = 0.39). There was no significant association between being *Cryptosporidium-*positive and having an HIV-positive biological mother (n = 1,194 tested; OR = 1.17; 95% CI: 0.77–1.77).

### Breastfeeding

Most children under two years old (81.4%) were partially breastfed. Breastfeeding was similar between *Cryptosporidium-*positive and *Cryptosporidium-*negative cases (Table 4).

**Table 4:** Breastfeeding status of GEMS-Kenya cases <24 months of age (N = 1,083) by *Cryptosporidium* status, western Kenya, 2008–2011.

	*Cryptosporidium-*positive cases (N = 142)^a^	*Cryptosporidium-*negative cases (N = 941)^a^
Age 0–5 months	n = 26	n = 220
Exclusively breastfed	5 (19.2%)	49 (22.3%)
Partially breastfed	20 (76.9%)	165 (75.0%)
Not breastfed	1 (3.9%)	6 (2.7%)
Age 6–11 months	n = 69	n = 358
Exclusively breastfed	2 (2.9%)	4 (1.1%)
Partially breastfed	64 (92.8%)	332 (92.7%)
Not breastfed	3 (4.4%)	22 (6.2%)
Age 12–23 months	n = 47	n = 363
Exclusively breastfed	0	1 (0.3%)
Partially breastfed	37 (78.7%)	264 (72.7%)
Not breastfed	10 (21.3%)	98 (27.0%)

a. The denominator for those with available data on breastfeeding. Represents only children in first three years of GEMS as questions related to breastfeeding practices were not compatible during the fourth year of GEMS.

Partial breastfeeding refers to giving children breast milk in addition to other food or liquid, whereas exclusive breastfeeding refers to giving children only breast milk without supplementation.

### Hospitalizations and deaths

There was no significant difference in the proportion of *Cryptosporidium*-positive cases and *Cryptosporidium*-negative cases who were hospitalized at enrollment (13.3% vs. 10.5%, *p* = 0.24). Among those with 60-day follow-up information, 9 (4.8%) of 187 *Cryptosporidium*-positive cases and 53 (3.5%) of 1,531 *Cryptosporidium*-negative cases died by the time of follow-up (*p* = 0.35). The cause of death, as per verbal autopsy, for the 9 children who died and had *Cryptosporidium* identified in their stool was as follows: HIV/AIDS related (n = 5), diarrhea/gastroenteritis (n = 1), pneumonia (n = 1), and anemia (n = 1); one child did not have a verbal autopsy completed.

### Water, sanitation, and hygiene (WASH) characteristics

The most common caretaker-reported primary drinking water sources were rainwater (35%), surface water (31%), other improved water sources (23%), and other unimproved water sources (11%); (Table 5). Compared to cases in households that used rainwater as the primary source of drinking water, case children living in households using other improved sources or unimproved sources (other than surface water) had significantly higher odds of *Cryptosporidium* infection (OR = 1.72; 95% CI: 1.14–2.58 and 2.12; 95% CI: 1.31–3.41, respectively).

**Table 5:** Water, sanitation, and hygiene characteristics of GEMS-Kenya cases (N = 1,778) by *Cryptosporidium* status, western Kenya, 2008–2012.

	*Cryptosporidium-* positive cases (N = 195)	*Cryptosporidium-* negative cases (N = 1,583)	OR (95% CI)
**Primary source of drinking water**^**a**^			
Rainwater	51 (26.2%)	580 (36.6%)	ref.
Other improved water sources^b^	53 (27.2%)	351 (22.2%)	**1.72 (1.14, 2.58)**
Surface water^b^	60 (30.8%)	486 (30.7%)	1.40 (0.95, 2.08)
Other unimproved water sources^b^	31 (15.9%)	166 (10.5%)	**2.12 (1.31, 3.41)**
**Water availability and storage**			
Water always available from main source	178 (91.3%)	1,427 (90.1%)	1.14 (0.70, 2.00)
Gave child stored water in past 2 weeks	173 (88.7%)	1,441 (91.0%)	0.77 (0.49, 1.28)
**Drinking water treatment**^**c**^			
Boils or filters water:	10 (5.1%)	149 (9.4%)	**0.52 (0.25, 0.96)**
Boils	10	147	---
Ceramic water filter	0	2	---
Does not boil or filter water:	185 (94.9%)	1,434 (90.6%)	ref.
Chlorinates water	103	804	---
Other treatment method	5	53	---
Does not treat drinking water	77	577	---
**Sanitation facilities**			
No waste facility for feces disposal	29 (14.9%)	262 (16.6%)	0.88 (0.58, 1.34)
Has facility for feces disposal	166 (85.1%)	1,321 (83.4%)	ref.
Traditional pit toilet	158	1,183	---
Ventilated improved pit latrine	7	102	---
Other (flush, pour/flush, or other facility)	1	36	---
**Hand hygiene**^**d**^			
Washes hands before eating	162 (83.1%)	1,318 (83.3%)	0.99 (0.67, 1.49)
Washes hands after defecating	141 (72.3%)	1,234 (78.0%)	0.74 (0.53, 1.04)
Washes hands before nursing	63 (32.3%)	475 (30.0%)	1.11 (0.81, 1.53)
Washes hands before cooking	57 (29.2%)	538 (34.0%)	0.80 (0.58, 1.11)
Washes hands after cleaning child	56 (28.7%)	405 (25.6%)	1.17 (0.84, 1.63)
Washes hands after touching animal	18 (9.2%)	184 (11.6%)	0.77 (0.45, 1.25)
Uses soap when washing hands	181 (92.8%)	1,491 (94.2%)	0.80 (0.45, 1.43)

OR = odds ratio (logistic regression); CI = confidence interval; ref. = referent group; **bolding** indicates statistically significant at *p*<0.05

a. In the two weeks prior to enrollment; as reported by caretaker

b. Other improved water sources: water piped into the house/yard, public taps, deep tube wells, covered wells, protected springs, or boreholes. Surface water sources: pond, lake, river, stream, dam, or earth pan/water pan. Other unimproved water sources: open wells, shallow tube wells, unprotected springs, and purchased water (such as bottled water).

c. Caretakers were asked what method they used most often when treating water at home

d. Caretakers were asked to list times they usually wash their hands without probing from the questionnaire administrators

Few caretakers of GEMS-Kenya case children reported boiling or using a ceramic filter to treat drinking water; however, those reporting one of these methods had significantly lower odds of *Cryptosporidium* infection compared to those who didn’t (OR = 0.52, 95% CI: 0.25–0.96), predominantly driven by those who boiled water. Only two households reported filtering.

Reported handwashing behavior was similar among the caretakers of *Cryptosporidium*-positive and *Cryptosporidium*-negative cases (Table 5).

Only age category remained in the final multivariable demographic, environmental, and behavioral characteristics model; thus, this model is not presented.

### *Cryptosporidium* genotyping

DNA was extracted from a random subset of 64 (40%) of the 160 *Cryptosporidium*-positive stool specimens from GEMS-Kenya case children enrolled in the first three years. Nested 18S PCR detected *Cryptosporidium* in 43 (67%) of these specimens. Of the 43 specimens, 35 (81%) were of the species *C*. *hominis* and 6 (14%) were *C*. *parvum*. *C*. *meleagridis* and *C*. *canis* were found in one specimen each.

### Sensitivity analysis

**Fig 2:**
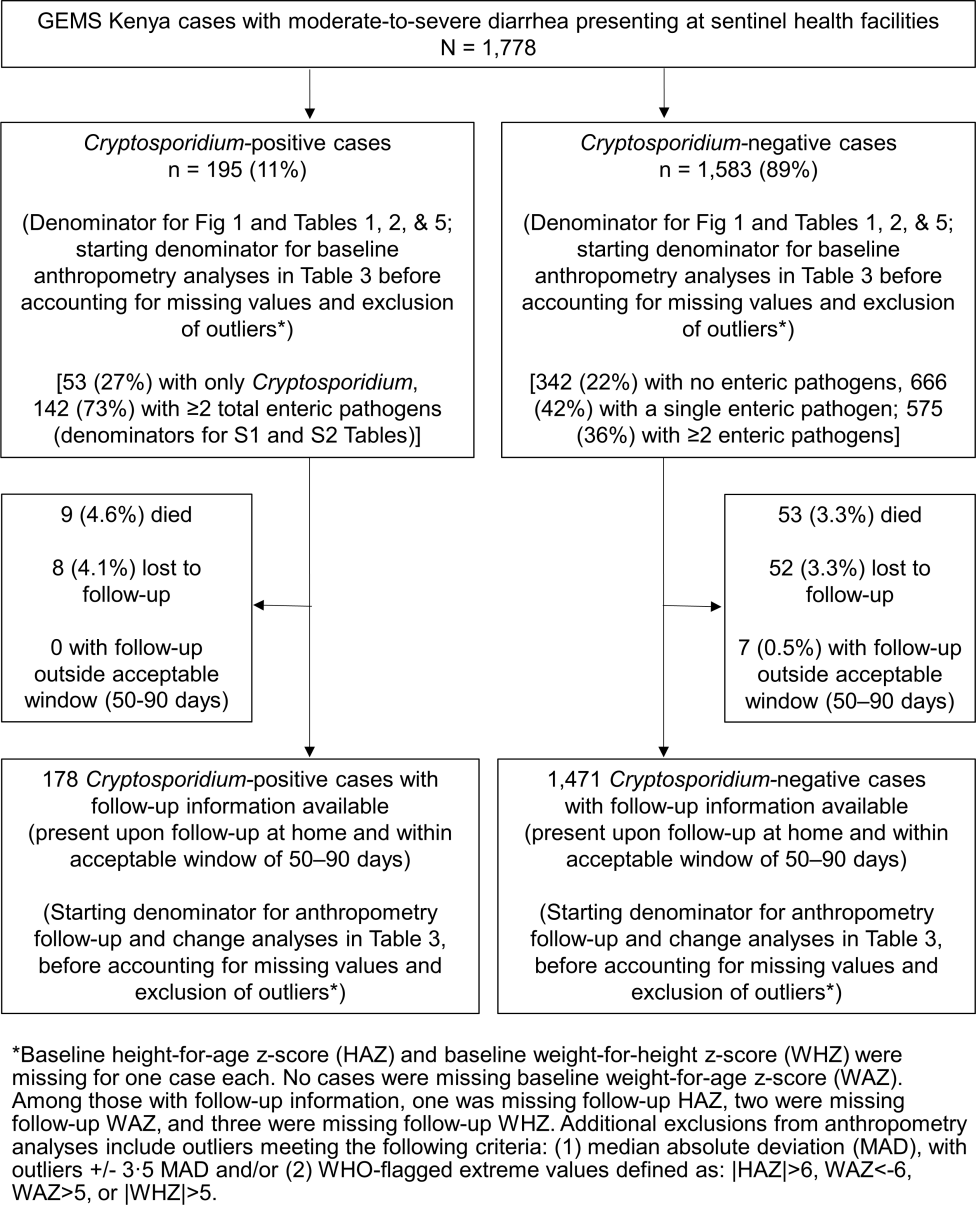
GEMS-Kenya cases (N = 1,778) with *Cryptosporidium* status and follow-up status/anthropometric measurements, western Kenya, 2008–2012.

Of the 195 *Cryptosporidium*-positive cases, 142 (72.8%) also had one or more additional enteric pathogens identified in their stool; enteric co-infections were common throughout the study population (Fig 2). The characteristics of case children with only *Cryptosporidium* detected in their stool were generally similar to *Cryptosporidium*-positive case children with multiple enteropathogens (S1 and S2 Tables); the only significant clinical difference was in the child’s mental state at enrollment (S1 Table). Variables chosen for multivariable models were unchanged when excluding children who were enrolled more than once as an MSD case.

## Discussion

This study evaluated the clinical, environmental, and behavioral characteristics associated with *Cryptosporidium* infection among children under five years old with MSD in rural western Kenya. Overall, 11% of children with MSD had *Cryptosporidium* identified in their stool; the majority (81%) of genotyped samples were *C*. *hominis*. Among MSD cases, being an infant, having mucus in stool, and having prolonged or persistent duration diarrhea were associated with being *Cryptosporidium-*positive. Boiling drinking water and using rainwater as the main drinking water source appeared to protect against *Cryptosporidium* infection in MSD cases. Among girls, *Cryptosporidium-*positive cases were more likely to be stunted at baseline compared to *Cryptosporidium-*negative cases. *Cryptosporidium-*positive cases had longer-term consequences in terms of malnutrition, as these children were more likely to stunted, underweight, or wasted at follow-up (controlling for baseline status), and have significantly larger negative changes in height- and weight-for-age z-scores.

Except for having mucus in stool, which could be associated with *Cryptosporidium* adhering to the small intestine mucosa, possibly causing inflammation [29], the clinical presentation of children with MSD was similar for *Cryptosporidium*-positive and *Cryptosporidium*-negative cases, as was observed in another study of Kenyan children with diarrhea [16]. This finding highlights the difficulty in clinically diagnosing *Cryptosporidium* among children with MSD in this setting and underscores the need for point of care rapid diagnostics for *Cryptosporidium*.

Infants were over three times more likely to have *Cryptosporidium* identified in their stool compared with children aged 24–59 months. The peak of infection at 6–11 months in this study is similar to the age pattern of *Cryptosporidium* infections previously reported in sub-Saharan Africa, though an earlier peak than other studies in Kenya [9,16]. This timeframe may coincide with the introduction of complementary foods or drinking water. The high prevalence of *Cryptosporidium* infections in young children is concerning as *Cryptosporidium* infections in early childhood have been associated with numerous poor outcomes, sometimes lasting beyond the initial infection [6,7], as evident in our findings.

Prolonged and persistent duration diarrhea, and growth shortfalls subsequent to enrollment were significantly more pronounced among *Cryptosporidium-*positive cases compared to other children with MSD. Prolonged and persistent diarrheal episodes occurring in infants have been previously associated with growth shortfalls [30]. The proportion of *Cryptosporidium*-positive cases who were underweight and wasted increased from baseline to follow-up. This could result from many days of diarrhea experienced by these children. There was also an increase in the proportion of *Cryptosporidium*-positive cases who were stunted from baseline (29%) to follow-up (39%). Undernutrition and stunting among children in low- and middle-income countries have predicted decreased performance in school and on cognitive tests in previous research [31], thus even longer-term consequences could be appreciable although unexplored in the current study. It is estimated that growth faltering contributes substantially to the overall global burden of disease from *Cryptosporidium* infections in children [4]. By the time of follow-up, 4.8% of *Cryptosporidium-*positive and 3.5% of *Cryptosporidium-*negative cases had died. Although the difference was not statistically significant, this warrants close future attention since other research has shown an association between *Cryptosporidium* and excess mortality for children who became infected in infancy [5].

Like other studies in Kenya [9,16,32], our findings indicate that person-to-person transmission is likely the predominant route for *Cryptosporidium* infection in rural western Kenya, since the main host for *C*. *hominis* is humans [10]. Infections may thus more commonly result from exposure to human feces than animal feces. The presence of animals in the compound was examined in univariable analyses but was not reported in detail or included in the risk factor model selection, as the ownership of many types of animals was not associated with *Cryptosporidium* infection (S3 Table), though it may be associated with unmeasured confounders (*e*.*g*., higher income). Notably, in another study, *C*. *hominis* was associated with more severe clinical symptoms in Kenyan children compared to *C*. *parvum* [32], although we have too little data in GEMS Kenya to examine this.

Using rainwater as the main drinking water source was common and was significantly protective against *Cryptosporidium* infections. Rainwater may be less contaminated with *Cryptosporidium*, or this finding could be related to the seasonality of *Cryptosporidium* infections. The proportion of households using rainwater as the main drinking water source varied by month, ranging from 3%-74%. We did not explore *Cryptosporidium* infections by month/season, as the biweekly enrollment targets for GEMS make interpretation of pathogen-specific seasonal analyses challenging. However, the fact that using rainwater as the primary drinking water source and boiling drinking water were both protective against *Cryptosporidium* infections indicates that drinking water source choices and certain treatment options may be effective in reducing *Cryptosporidium* infections and signals that water may play a role in transmission.

A limitation of this work is that a comparison could not be made between *Cryptosporidium-*positive GEMS cases and GEMS controls, as reliable population weights were not available at the time of analysis. The factors associated with *Cryptosporidium* compared to other individuals with MSD may be different from those risk factors that would be seen when compared to healthy controls. Using MSD as a condition for inclusion for our study may lead to spurious associations, as it is potentially a common effect of both the exposures and the outcome of interest [33]. Our ability to explore data on those with only *Cryptosporidium* infections was limited due to the small number of single-pathogen *Cryptosporidium* infections; however, those who presented with *Cryptosporidium* alone had similar characteristics to those who presented with multiple-pathogen *Cryptosporidium* infections. We were not able to assess the association between breastfeeding and *Cryptosporidium* because of (1) the collinearity between breastfeeding and age, and (2) the small number of children who were either exclusively or not breastfed in certain age groups. We also could not examine anthropometric outcomes by age or by the number of enteric pathogens isolated in stool, due to the small number of *Cryptosporidium*-positive children in some age groups and the small number of cases who presented with single-pathogen *Cryptosporidium* infections. We examined HIV status and malnutrition in our analyses, and performed a sensitivity analysis related to enteric co-infections; however, we did not have information on other co-morbidities that may be associated with *Cryptosporidium* infection.

The burden of diarrhea attributable to *Cryptosporidium* differed between GEMS and MAL-ED, especially for children 1–2 years old; however, GEMS generally considered more severe cases of diarrhea than MAL-ED. Other differences between the studies have been described elsewhere [34]. While GEMS and MAL-ED found *Cryptosporidium* to be significantly associated with diarrhea among infants, there were differences between study sites; in other studies *Cryptosporidium* has been isolated in non-diarrheal stools as often as in diarrheal stools [35,36]. Dissimilarities in study design (*e*.*g*., the time from diarrhea onset to stool collection) or laboratory methods may partially explain the differences observed, and host susceptibility and other risk factors are likely to vary across settings [35]. However, *Cryptosporidium* infections have been associated with growth shortfalls in asymptomatic children without diarrhea, thus identification and treatment of *Cryptosporidium* should remain a priority for young children in settings where it is endemic [35,36].

The high prevalence of cryptosporidiosis among young children in our study, coupled with other research that shows extended long-term effects of *Cryptosporidium* infections and diarrhea early in life, underscores the need for preventive measures aimed at households with young children, as well as improved diarrhea case management. Early diagnosis and management of cryptosporidiosis may mitigate subsequent growth deficits and other long-term consequences. Increased availability of nitazoxanide or new treatments, point of care rapid diagnostics for *Cryptosporidium*, additional insights into the role of appropriate WASH practices and technologies in childhood cryptosporidiosis, and vaccine development could reduce the burden of disease in such settings. Since *Cryptosporidium-*positive cases experienced more days of diarrhea and subsequent malnutrition than other MSD cases, increased promotion of the use of zinc in the management of diarrhea, and continued feeding of children with diarrhea should be undertaken, per WHO/UNICEF guidelines [37]. As rotavirus vaccine coverage increases, potentially leading to an altered enteric pathogen landscape, continuing to examine the impact and relative importance of *Cryptosporidium* infection among infants should remain a priority.

## Supporting information

S1 Checklist STROBE checklist for observational study; checklist for analysis of factors associated with *Cryptosporidium* infection in cases enrolled in the GEMS case-control study, western Kenya, 2008–2012. (DOC)

S1 Table Comparison of *Cryptosporidium*-positive GEMS-Kenya cases (N = 195) with a single pathogen to *Cryptosporidium*-positive GEMS-Kenya cases with multiple pathogens: clinical variables, western Kenya, 2008–2012. (DOCX)

S2 Table Comparison of *Cryptosporidium*-positive GEMS-Kenya cases (N = 195) with a single pathogen to *Cryptosporidium*-positive GEMS-Kenya cases with multiple pathogens: exposure/risk factor variables, western Kenya, 2008–2012. (DOCX)

S3 Table Presence of animals in the compounds of *Cryptosporidium*-positive and *Cryptosporidium*-negative GEMS-Kenya cases (N = 1,778), western Kenya, 2008–2012. (DOCX)
