# Supplementary material for: Clinical, environmental, and behavioral characteristics associated with Cryptosporidium infection among children with moderate-to-severe diarrhea in rural western Kenya, 2008–2012: The Global Enteric Multicenter Study (GEMS)
Source: PLoS Negl Trop Dis. 2018 Jul 12;12(7):e0006640. doi: 10.1371/journal.pntd.0006640 (PMC6057667; doi:10.1371/journal.pntd.0006640)
Supplement: S1 Checklist — (DOC) [file pntd.0006640.s001.doc]

**S1 Checklist. STROBE checklist for observational study; checklist for analysis of factors associated with *Cryptosporidium* infection in cases enrolled in the GEMS case-control study, western Kenya, 2008-2012**

|  | | | Item No | Recommendation | Section | Paragraph within section | |
| --- | --- | --- | --- | --- | --- | --- | --- |
| **Title and abstract** | | | 1 | (*a*) Indicate the study’s design with a commonly used term in the title or the abstract | abstract | paragraph 2 (Methodology/Principal Findings section) | |
|  |  |  |  | (*b*) Provide in the abstract an informative and balanced summary of what was done and what was found | abstract | paragraphs 1-3 | |
| Introduction | | | | |  |  | |
| Background/rationale | | | 2 | Explain the scientific background and rationale for the investigation being reported | Introduction | paragraphs 1-4 | |
| Objectives | | | 3 | State specific objectives, including any prespecified hypotheses | Introduction | paragraph 5 | |
| Methods | | | | |  |  | |
| Study design | | | 4 | Present key elements of study design early in the paper | Global Enteric Multicenter Study (GEMS) | paragraph 1 | |
| Setting | | | 5 | Describe the setting, locations, and relevant dates, including periods of recruitment, exposure, follow-up, and data collection | Study Site | paragraph 1 | |
| Participants | | | 6 | (*a*) Give the eligibility criteria, and the sources and methods of case ascertainment and control selection. Give the rationale for the choice of cases and controls | Global Enteric Multicenter Study (GEMS) | paragraph 1 (and supporting GEMS citations) | |
|  |  |  |  | (*b*) For matched studies, give matching criteria and the number of controls per case |  |  |  |
| Variables | | | 7 | Clearly define all outcomes, exposures, predictors, potential confounders, and effect modifiers. Give diagnostic criteria, if applicable | (1) Global Enteric Multicenter Study (GEMS)  (2) Study definitions | (1) paragraph 2 (*Cryptosporidium* diagnostic criteria)  (2) paragraphs 1-5 | |
| Data sources/ measurement | | | 8* | For each variable of interest, give sources of data and details of methods of assessment (measurement). Describe comparability of assessment methods if there is more than one group | Study definitions | paragraphs 1-5 | |
| Bias | | | 9 | Describe any efforts to address potential sources of bias | Sensitivity analyses | paragraph 1 | |
| Study size | | | 10 | Explain how the study size was arrived at | Global Enteric Multicenter Study (GEMS) | paragraph 1 (supporting GEMS citations) | |
| Quantitative variables | | | 11 | Explain how quantitative variables were handled in the analyses. If applicable, describe which groupings were chosen and why | (1) Study definitions  (2) Anthropometry analyses | (1) paragraphs 1-2  (2) paragraph 1 | |
| Statistical methods | | | 12 | (*a*) Describe all statistical methods, including those used to control for confounding | Statistical analysis | paragraphs 1-2 | |
|  |  |  |  | (*b*) Describe any methods used to examine subgroups and interactions | (1) Statistical analysis  (2) Anthropometry analyses | (1) paragraph 1  (2) paragraph 1 | |
|  |  |  |  | (*c*) Explain how missing data were addressed | Fig 2; Tables 1-5 | Fig 2 footnote; tables can be used to confirm data are not missing in other analyses | |
|  |  |  |  | (*d*) If applicable, explain how matching of cases and controls was addressed | n/a |  | |
|  |  |  |  | (*e*) Describe any sensitivity analyses | Sensitivity analyses | paragraph 1 | |
| Results | | | | |  |  | |
| Participants | | | 13* | (a) Report numbers of individuals at each stage of study—eg numbers potentially eligible, examined for eligibility, confirmed eligible, included in the study, completing follow-up, and analysed | Fig 2 | n/a | |
|  |  |  |  | (b) Give reasons for non-participation at each stage | Fig 2 | n/a | |
|  |  |  |  | (c) Consider use of a flow diagram | Fig 2 | n/a | |
| Descriptive data | | | 14* | (a) Give characteristics of study participants (eg demographic, clinical, social) and information on exposures and potential confounders | Tables 1-2 | n/a | |
|  |  |  |  | (b) Indicate number of participants with missing data for each variable of interest | Fig 2; Tables 1-5 | Fig 2 footnote; tables can be used to confirm data are not missing in other analyses | |
| Outcome data | | | 15* | Report numbers in each exposure category, or summary measures of exposure | Demographic and household characteristics | paragraph 1 | |
| Main results | | | 16 | (*a*) Give unadjusted estimates and, if applicable, confounder-adjusted estimates and their precision (eg, 95% confidence interval). Make clear which confounders were adjusted for and why they were included | Results | paragraph 1-11 | |
|  |  |  |  | (*b*) Report category boundaries when continuous variables were categorized | Tables 1-3; Fig 1 | n/a | |
|  |  |  |  | (*c*) If relevant, consider translating estimates of relative risk into absolute risk for a meaningful time period | n/a |  | |
| Other analyses | 17 | Report other analyses done—eg analyses of subgroups and interactions, and sensitivity analyses | | | (1) Indicators of malnutrition  (2) HIV status  (3) Breastfeeding  (4) *Cryptosporidium* genotyping  (5) Sensitivity analyses  (6) Fig 2; Table 3; S1-S2 Tables | | (1) paragraph 1  (2) paragraph 1  (3) paragraph 1  (4) paragraph 1  (5) paragraph 1  (6) n/a |
| Discussion | | | | |  | |  |
| Key results | 18 | Summarise key results with reference to study objectives | | | Discussion | | paragraph 1 |
| Limitations | 19 | Discuss limitations of the study, taking into account sources of potential bias or imprecision. Discuss both direction and magnitude of any potential bias | | | Discussion | | paragraph 7 |
| Interpretation | 20 | Give a cautious overall interpretation of results considering objectives, limitations, multiplicity of analyses, results from similar studies, and other relevant evidence | | | Discussion | | paragraph 2-6; paragraph 8 |
| Generalisability | 21 | Discuss the generalisability (external validity) of the study results | | | Discussion | | paragraph 8 |
| Other information | | | | |  | |  |
| Funding | 22 | Give the source of funding and the role of the funders for the present study and, if applicable, for the original study on which the present article is based | | | Online funding statement | | n/a |

Source of STROBE recommendations (https://www.strobe-statement.org/fileadmin/Strobe/uploads/checklists/STROBE_checklist_v4_case-control.pdf)
