# Supplementary material for: Clinical, environmental, and behavioral characteristics associated with Cryptosporidium infection among children with moderate-to-severe diarrhea in rural western Kenya, 2008–2012: The Global Enteric Multicenter Study (GEMS)
Source: PLoS Negl Trop Dis. 2018 Jul 12;12(7):e0006640. doi: 10.1371/journal.pntd.0006640 (PMC6057667; doi:10.1371/journal.pntd.0006640)
Supplement: S1 Table — (DOCX) [file pntd.0006640.s002.docx]

**S1 Table. Comparison of *Cryptosporidium*-positive GEMS-Kenya cases (N=195) with a single pathogen to *Cryptosporidium*-positive GEMS-Kenya cases with multiple pathogens: clinical variables, western Kenya, 2008-2012**

|  | ***Cryptosporidium-*positive cases:**  **single pathogen only**  (N=53) | ***Cryptosporidium-*positive cases: multiple pathogens**  (N=142) | ***p*-value^a^** |
| --- | --- | --- | --- |
| **Measured or observed by clinician at enrollment** |  |  |  |
| Had fever at enrollment^b^ | 13 (24.5%) | 37 (26.1%) | 0.83 |
| Required intravenous rehydration | 6 (11.3%) | 27 (19.0%) | 0.20 |
| Child’s mouth: |  |  | 0.43^F^ |
| Normal | 3 (5.7%) | 3 (2.1%) |  |
| Somewhat dry | 40 (75.5%) | 108 (76.1%) |  |
| Very dry | 10 (18.9%) | 31 (21.8%) |  |
| Child’s mental state: |  |  | **0.01^F^** |
| Normal | 26 (49.1%) | 38 (26.8%) |  |
| Irritable/restless | 26 (49.1%) | 99 (69.7%) |  |
| Lethargic or Unconscious | 1 (1.9%) | 5 (3.5%) |  |
| **Reported by caretaker at enrollment** |  |  |  |
| Child is very thirsty | 43 (81.1%) | 110 (77.5%) | 0.58 |
| Child drinks poorly/is unable to drink | 10 (18.9%) | 22 (15.5%) | 0.57 |
| Child has wrinkled skin | 9 (17.0%) | 30 (21.1%) | 0.52 |
| Child has fast breathing | 9 (17.0%) | 18 (12.7%) | 0.44 |
| Maximum # of stools child passed in a 24-hour period during illness up to enrollment |  |  | 0.65^F^ |
| ≤6 | 42 (79.2%) | 103 (72.5%) |  |
| 7-10 | 9 (17.0%) | 33 (23.2%) |  |
| >10 | 2 (3.8%) | 6 (4.2%) |  |
| **Characteristics of stool sample provided at enrollment** |  |  |  |
| Stool sample watery^c^ | 26 (49.1%) | 88 (62.0%) | 0.10 |
| Blood in stool sample | 0 (0%) | 6 (4.2%) | n/a |
| Pus in stool sample | 3 (5.7%) | 4 (2.8%) | 0.39^F^ |
| Mucus in stool sample | 39 (73.6%) | 116 (81.7%) | 0.21 |
| F: Fisher’s exact test used; **bolding** indicates statistically significant at *p*<0.05  a. *p*-value of significant difference between columns  b. Temperature >38°C measured in health facility  c. Compared to stool that was formed, soft, or thick liquid | | | |
