# Supplementary material for: Clinical, environmental, and behavioral characteristics associated with Cryptosporidium infection among children with moderate-to-severe diarrhea in rural western Kenya, 2008–2012: The Global Enteric Multicenter Study (GEMS)
Source: PLoS Negl Trop Dis. 2018 Jul 12;12(7):e0006640. doi: 10.1371/journal.pntd.0006640 (PMC6057667; doi:10.1371/journal.pntd.0006640)
Supplement: S2 Table — (DOCX) [file pntd.0006640.s003.docx]

**S2 Table. Comparison of *Cryptosporidium*-positive GEMS-Kenya cases (N=195) with a single pathogen to *Cryptosporidium*-positive GEMS-Kenya cases with multiple pathogens: exposure/risk factor variables, western Kenya, 2008-2012**

|  | ***Cryptosporidium-*positive cases:**  **single pathogen only**  (N=53) | ***Cryptosporidium-*positive cases:**  **multiple pathogens**  (N=142) | ***p*-value^a^** |
| --- | --- | --- | --- |
| Age Category |  |  | 0.75 |
| Age 0-11 months | 30 (56.6%) | 89 (62.7%) |  |
| Age 12-23 months | 18 (34.0%) | 36 (25.4%) |  |
| Age 24-59 months | 5 (9.4%) | 17 (12.0%) |  |
| Male sex | 22 (41.5%) | 53 (37.3%) | 0.59 |
| Caretaker completed primary school | 26 (49.1%) | 70 (49.3%) | 0.98 |
| People sleeping in house >median^b^ | 18 (34.0%) | 64 (45.1%) | 0.16 |
| Young children^c^ in house >median^b^ | 6 (11.3%) | 24 (16.9%) | 0.34 |
| House has agricultural land | 49 (92.5%) | 124 (87.3%) | 0.32 |
| Main source of drinking water^d^ |  |  | 0.68 |
| Rainwater | 13 (24.5%) | 38 (26.8%) |  |
| Other improved water | 11 (20.8%) | 42 (29.6%) |  |
| Surface water | 18 (34.0%) | 42 (29.6%) |  |
| Other unimproved water | 11 (20.8%) | 20 (14.1%) |  |
| Water always available from main source | 48 (90.6%) | 130 (91.5%) | 0.83 |
| Gave child stored water in past 2 weeks | 49 (92.5%) | 124 (87.3%) | 0.45^F^ |
| Boils or filters water | 1 (1.9%) | 9 (6.3%) | 0.29^F^ |
| No waste facility for feces disposal | 3 (5.7%) | 26 (18.3%) | **0.04^F^** |
| Washes hands before eating | 45 (84.9%) | 117 (82.4%) | 0.68 |
| Washes hands after defecating | 37 (69.8%) | 104 (73.2%) | 0.64 |
| Washes hands before nursing | 18 (34.0%) | 45 (31.7%) | 0.76 |
| Washes hands before cooking | 14 (26.4%) | 43 (30.3%) | 0.60 |
| Washes hands after cleaning child | 10 (18.9%) | 46 (32.4%) | 0.06 |
| Washes hands after touch animal | 3 (5.7%) | 15 (10.6%) | 0.41^F^ |
| Uses soap when washing hands | 48 (90.6%) | 133 (93.7%) | 0.53^F^ |
| F: Fisher’s exact test used; **bolding** indicates statistically significant at *p*<0.05  a. *p*-value of significant difference between columns  b. Above median of all GEMS-Kenya cases combined; people sleeping in house determined by caretaker response to “how many people have been sleeping regularly in your household for the past 6 months?”  c. Young children are defined as children <5 years old  d. Other improved water sources: water piped into the house/yard, public taps, deep tube wells, covered wells, protected springs, or boreholes. Surface water sources: pond, lake, river, stream, dam, or earth pan/water pan. Other unimproved water sources: open wells, shallow tube wells, unprotected springs, and purchased water (such as bottled water). | | | |
