# Supplementary material for: Clinical, environmental, and behavioral characteristics associated with Cryptosporidium infection among children with moderate-to-severe diarrhea in rural western Kenya, 2008–2012: The Global Enteric Multicenter Study (GEMS)
Source: PLoS Negl Trop Dis. 2018 Jul 12;12(7):e0006640. doi: 10.1371/journal.pntd.0006640 (PMC6057667; doi:10.1371/journal.pntd.0006640)
Supplement: S3 Table — (DOCX) [file pntd.0006640.s004.docx]

**S3 Table. Presence of animals in the compounds of *Cryptosporidium*-positive and *Cryptosporidium*-negative GEMS-Kenya cases (N=1,778), western Kenya, 2008-2012**

| **Animal** | ***Cryptosporidium-***  **positive cases** (N=195) | ***Cryptosporidium-***  **negative cases** (N=1,583) |
| --- | --- | --- |
| Goat | 96 (49.2%) | 894 (56.5%) |
| Cow | 119 (61.0%) | 1,044 (66.0%) |
| Sheep | 54 (27.7%) | 484 (30.6%) |
| Fowl | 183 (93.8%) | 1,495 (94.4%) |
| Dog | 123 (63.1%) | 1,013 (64.0%) |
| Cat | 136 (69.7%) | 1,056 (66.7%) |
| Rodents | 92 (47.2%) | 869 (54.9%) |
| Other Animal | 13 (6.7%) | 86 (5.4%) |
